# Plexiform fibromyxoma: Case report and literature review

**DOI:** 10.1097/MD.0000000000027164

**Published:** 2021-09-10

**Authors:** Min Lin, Lu Song, Shuming Qin, Daosheng Li, Gang Hou, Xiaomei Li

**Affiliations:** a Department of Pathology, Tai’an City Central Hospital, Tai’an, China; b Department of Breast Surgery, Tai’an City Central Hospital, Tai’an, China.

**Keywords:** gastric adenocarcinoma, gastrointestinal stromal tumor, plexiform fibromyxoma, synchronous tumors

## Abstract

Plexiform fibromyxoma (PF) is a rare mesenchymal neoplasm which can be misdiagnosed as the gastrointestinal stromal tumor. This tumor almost formed a lobulated intramural/submucosal mass in the gastric antrum and prepyloric area. It was considered as a benign tumor that exhibited no recurrence, metastasis, or tumor-related mortality. In this study, we reported 2 cases of gastric PF. The first case was a PF patient coexisting with gastric adenocarcinoma. The second case occurred in the gastric upper body close to gastric fundus. They underwent distal gastrectomy and laparoscopic partial gastric resection, respectively. Both of them exhibited a plexiform growth pattern in the submucosa, muscularis propria, and subserosal adipose tissues. The nodules were composed of abundant myxoid or fibromyxoid matrix riching in small thin-walled blood vessels and bland-looking spindle cells. The first case partially showed staggered growth pattern of PF and adenocarcinoma. Immunohistochemically, the spindle cells were diffusely immunoreactive for SMA and vimentin, and focally immunoreactive for CD10. It was important to distinguish the PF from other spindle cell tumors involving the stomach.

## 1 Introduction

Gastrointestinal stromal tumor (GIST) is the most common gastric mesenchymal tumor affecting the public health worldwide.^[1]^ Typically, more than 90% of the GIST cases showed expression of CD117 and DOG-1,^[2]^ while more than 80% of the cases expressed CD34.^[3]^

Plexiform fibromyxoma (PF), also known as plexiform angiomyxoidmyofibroblastic tumor, is a rare benign mesenchymal neoplasm of the stomach. It was first described in 2007^[4]^ and officially designated by the World Health Organization in 2010.^[5]^ Most PF is arisen from the antrum and pyloric region, forming a lobulated intramural/submucosal mass. In clinical settings, PF is often misdiagnosed as GIST due to similar clinical manifestations. Rare cases showed synchronous occurrence of PF and other primary gastrointestinal tumors. In this study, we reported 2 cases with PF in stomach, together with describing the clinical characteristics, histopathologic and immunophenotypical features, as well as the discussion on the misleading differential diagnosis. More importantly, among the 2 cases, 1 showed co-existence of PF and gastric adenocarcinoma that had never reported in the previous literatures.

## 2 Case presentation

### 2.1 Case 1

A 64-year-old female patient presented to our department due to epigastric discomfort for 7 days. Laboratory findings were normal. Computed tomography (CT) scan and endoscopic ultrasound demonstrated a solid mass within the submucosa and muscularis propria of the gastric antrum. Endoscopic biopsy showed epithelial dysplasia in focal mucosa, and possibility of gastric adenocarcinoma should not be excluded. On this basis, distal gastrectomy was conducted. Grossly, the mass (2.5 cm × 2 cm × 1.7 cm) was localized at the antrum close to the pylorus region with a mucosa ulceration (1 cm × 1 cm). The tumor mass was well demarcated with a solid nature and mucoid appearance. Its growth was in a multinodular pattern, mainly involving the submucosa, muscularis propria, and subserosal adipose tissues.

**Figure 1:**
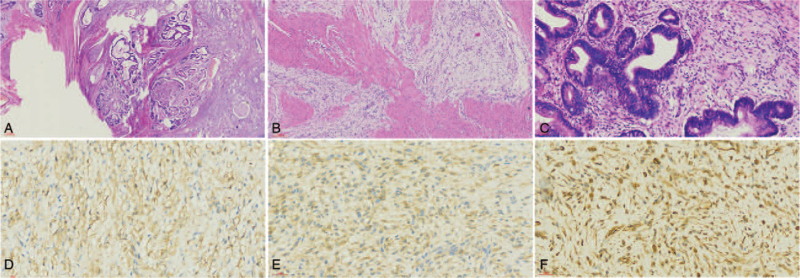
Histopathological and immunohistochemical findings for case 1. (A) Dysplasia glands infiltrated the mucosa to muscularis propria after hematoxylin and eosin staining under a magnification of 20×. (B) Plexiform growth pattern involved the submucosa, muscularis propria, and subserosal adipose tissues. The nodules consisted of fibromyxoid matrix which was rich in small thin-walled blood vessels and bland-looking spindle cells after HE staining under a magnification of 100×. (C) Staggering growth pattern of adenocarcinoma and PF. HE staining, 200×. (D–F) Immunoreaction for SMA, CD10, and Vim of the focal spindle cells under a magnification of 400×. PF = plexiform fibromyxoma.

For the histopathologic findings, 2 different morphological manifestations were observed as follows: The dysplasia glands infiltrated the mucosa to muscularis propria. On this basis, moderately differentiated adenocarcinoma was defined (Fig. 1A). A plexiform growth pattern involved the submucosa, muscularis propria, and subserosal adipose tissue. The nodules were composed of fibromyxoid matrix which was rich in small thin-walled blood vessels and bland-looking spindle cells (Fig. 1B). The spindle tumor cells showed no significant nuclear atypia, mitotic activity, and necrosis. The gastric adenocarcinoma and PF partially showed staggered growth pattern without lymphatic metastasis (Fig. 1C). Immunohistochemically, the spindle cells were diffusely immunoreactive for SMA (Fig. 1D) and vimentin (Fig. 1F). Additionally, the cells were focally immunoreactive for CD10 (Fig. 1E), and negative for CD117, DOG1, CD34, S100, β-catenin, STAT-6, and ALK. The patient was followed up for 51 months, with no evidence of recurrence and metastasis.

### 2.2 Case 2

A 39-year-old male patient was admitted to our hospital for routine gastroscopy, which revealed an elevated mucosal lesion (2.0 cm × 2.0 cm) with ulceration in the gastric upper body. CT scan showed a mass within the wall of the gastric upper body close to gastric fundus. Laboratory findings were within the normal ranges. Physical examination on the abdomen was unremarkable. Endoscopic biopsy showed no malignant cells and endoscopic ultrasound-guided fine-needle aspiration biopsy showed bland-looking spindle cells. On this basis, partial gastric resection was performed under laparoscopic assistance as he was suspected with GIST.

**Figure 2:**
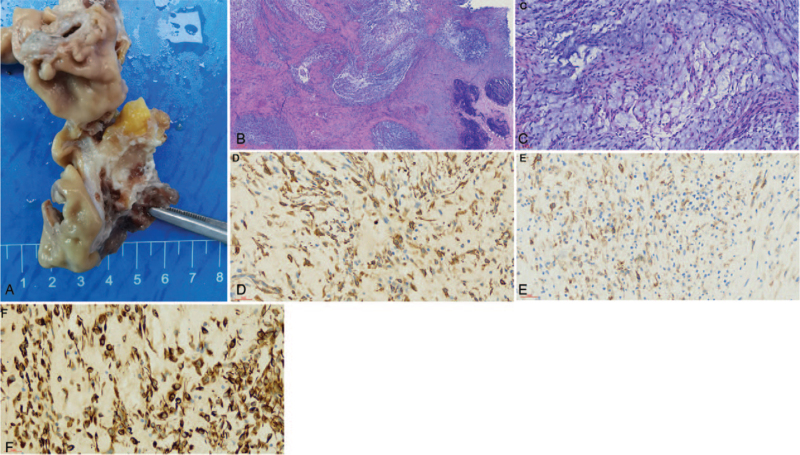
Pathological, histopathologic findings and immunohistochemical findings for case 2. (A) The cut surface was solid and mucoid. Multinodular growth pattern mostly involved the submucosa, muscularis propria, and subserosal adipose tissue. (B) Multiple intramural to subserosal nodules with characteristic plexiform growth with lymphoid tissue hyperplasia and lymphoid follicular formation (40×). (C) The tumor nodules contained a prominent myxoid stroma with low cellularity composed of bland spindle cells and delicate blood vessels surrounded the tumor cells (200×). (D–F) Immunoreaction for SMA, CD10, and Vim of the focal spindle cells (400×).

A multinodular growth pattern was noticed mainly involving the submucosa, muscularis propria, and subserosal adipose tissue. The tumor was well demarcated and the cut surface was solid and mucoid (Fig. 2A). Histology showed multiple intramural and subserosal nodules with characteristic plexiform growth with lymphoid tissue hyperplasia and lymphoid follicular formation (Fig. 2B). The tumor nodules contained a prominent myxoid stroma with sparse bland spindle cells (Fig. 2C). There were delicate blood vessels surrounded the tumor cells, which extended into the gastric mucosa causing ulceration. Besides, no necrosis was seen. Immunohistochemically, the spindle cells were diffusely immunoreactive for SMA (Fig. 2D) and vimentin (Fig. 2F). In addition, they were focally immunoreactive for CD10 (Fig. 2E) and negative for CD117, DOG1, CD34, S100, β-catenin, STAT-6, and anaplastic lymphoma kinase. The patient was followed up for 2 months with no recurrence and metastasis. The prognosis was satisfactory.

## 3 Discussion

PF is a rare gastric mesenchymal malignancy that has been reported among more than 100 cases. Takahashi et al^[4]^ first described 2 cases with plexiform angiomyxoidmyofibroblastic tumor in 2007. In 2009, Miettinen et al^[6]^ described 12 patients with PF. In 2010, this unique gastric mesenchymal tumor was defined as plexiformfibromyxoma by World Health Organization.^[4]^ Since then, more cases were reported all over the world.^[7–11]^

In this study, we reported 2 cases of PF in stomach, among which 1 showed coexistence of gastric adenocarcinoma. To our best knowledge, there was no such case before. Meanwhile, rare cases with simultaneous development of GIST and adenocarcinoma are available.^[12,13]^ As is known to all, stomach is the most common location for GIST with coexisting tumors^[14]^ and the major types of GIST-associated cancers are gastrointestinal carcinomas (47%).^[12]^ Due to rarity of the disease with tumors of different histotypes in the same organ, it is still not clear whether the synchronicity is accidental or related pathophysiological processes. Genetic mutations may be the basis for tumor predisposition in patients with 2 synchronous gastric tumors.^[13]^ In most cases with synchronous tumors, they presented separate lesions in different locations. In our case, an isolated lesion was noticed in which PF and adenocarcinoma showed staggered growth pattern causing challenges in the diagnosis.

The age of PF patients was in a range of 5 to 81 years with no gender differences.^[15]^ PF was commonly reported in adults and some pediatric patients^[16,17]^ with similar pathological features and benign clinical behaviors, except increased tumor growth in pediatric PF. This suggested that PF was the same disease entity regardless of age of onset in the previous description.^[18]^ The size of the tumors ranged from 0.8 cm to 17 cm in the maximal diameter (median: 4.0 cm).^[15]^ Although most PF is located in the gastric antrum and prepyloric area, the tumor could originate from the gastric body, duodenum, the jejunum, and the colon.^[11,19–21]^

PF patients typically present with abdominal discomfort and pain, anemia, and upper gastrointestinal bleeding, which are mainly caused by mucosal ulceration or other non-specific upper digestive tract symptoms. The diagnosis of PF is highly depending on pathological and immunochemical examinations, as the CT, magnetic resonance imaging, and gross examination findings of PF are similar with GIST. Mucosa ulcerations occurred in nearly half of the cases, which led to gastrointestinal hemorrhage and secondary anemia.^[7,11]^ The microscopic features of PF were relatively unique. These tumors often presented a multinodular growth pattern, involving the intra-mucosal to serosal of the stomach. The tumor nodules contained a prominent myxoid stroma rich in small thin-walled blood vessels with low to middle cellularity composed of bland spindle without cytological atypia, mitotic figures, or necrosis. In most areas, the tumor cells were arranged in a loose manner. The extracellular matrix within these tumors was usually positive by Alcian-blue staining. In some nodules, the collagenous stroma was densely hyalinized.^[7]^ Frequent ulceration, mucosal invasion, and vascular invasion had no adverse significance in these tumors.^[6]^ Immunohistochemically, the tumor cells were diffusely immunoreactive for SMA and vimentin, and focally immunoreactive for H-caldesmon, desmin, and CD10. The expression of myogenic markers may suggest that there were tumor cells with myofibroblastic-fibroblastic phenotype and true smooth muscle differentiation.^[22]^

The differential diagnosis of PF includes other spindle cell tumor involving the stomach. The tumor cells were negative for CD117, DOG1, CD34, S100, β-catenin, STAT-6, and ALK that played important roles in differential diagnosis. As the most common gastric mesenchymal tumors,^[1]^ GIST should be excluded initially. Few GISTs exhibited a plexiform and myxoid appearance similar to PF.^[10]^ However, GISTs typically expressed CD117, DOG-1, and CD34 and were negative for SMA, S-100, desmin, and AE1/AE3.^[2]^ In addition, 80% and 5% to 8% of GIST were primarily driven by the mutations in *KIT* and *PDGFRA*, respectively.^[23]^ These aspects were not seen in PF.

Myxoid leiomyoma, usually involving the esophagus, typically shows numerous spindle cells with cigar-shaped nuclei and eosinophilic cytoplasm. It contained no multinodular pattern, but was commonly seen in PF patients. Moreover, gastric leiomyoma was immunoreactive for desmin, actin, calponin, and h-caldesmon. Gastrointestinal schwannoma often formed a nodule lymphoid cuff with infiltrative margin and lymphoid cuff. They present diffuse immunoexpression of S-100 protein and SOX10. Inflammatory fibroid polyp occurred in a submucosal location. It was composed of bland short spindled cells which often arranged concentrically around vessels. There were many eosinophilic granulocytes in the loose fibromyxoid stroma. Most inflammatory fibroid polyp expressed CD34 and SMA rather than CD117, DOG-1, and S100. Inflammatory myofibroblastic tumor was composed of spindle myofibroblasts with lymphocytes and plasma cells in the background with intermediate biological behavior. In some cases, there might be occurrence of myxoid, vascular, and inflammatory areas resembling nodular fasciitis.^[24]^ About 50% of cases harbored rearrangements of *ALK* gene. Fibromatosis usually arises in mesentery but less gastric involvement has been reported.^[25]^ It has an infiltrative growth pattern and comprises of myofibroblasts. In focal parts, there were loose storiform structures. About 75% to 90% of cases exhibited nuclear expression of β-catenin and SMA immunoexpression.^[26]^

To date, only few reports have investigated the molecular biology of PF. In the previous study, 5 cases (31.25%) showed *GLI1* activation within the Hh pathway, including 2 (12.5%) with *GLI1* amplification and 3 (19.75%) with *MALAT1-GLI1* oncogenic fusions.^[27]^ Banerjee et al^[28]^ reported the first report of recurrent *PTCH1* loss in PF based on next generation sequencing and proposed that targeted Hh pathway inhibition with SMO antagonists might represent a target for treating a subset of PF. However, no *KIT* mutations or *PDGFRA* mutations are identified in PF.

Nowadays, the distal gastrectomy or partial gastrectomy is the main treatment option for PF involving the stomach. In a previous study,^[29]^ laparoscopic endoscopic cooperative surgery can be appropriate in the diagnose and therapy of submucosal tumors. It can prevent the deformation of the stomach, especially the tumor is located near the pylorus. PF acts as a benign tumor with few cases showing recurrence or distant metastases after complete surgical resection.^[6]^ In this study, the prognosis of case 1 was depending on the adenocarcinoma factors as PF acted as a benign neoplasm. In future, further studies are required to investigate the potential causal relationship between these synchronous tumors.

In summary, PF is a rare mesenchymal tumor mainly occurring in the gastric antrum, which is mainly diagnosed based on histological and immunohistochemical findings. Most of PF patients showed a lobulated submucosal mass and were often misdiagnosed as GIST because of the similar clinical manifestations. In clinical practice, more attention should be paid to it as it can coexist with gastric adenocarcinoma, resulting in a poor prognosis in the patients.

## Author contributions

**Conceptualization:** Lu Song.

**Data curation:** Lu Song.

**Formal analysis:** Lu Song.

**Funding acquisition:** Shuming Qin.

**Investigation:** Shuming Qin.

**Methodology:** Shuming Qin.

**Software:** Daosheng Li.

**Supervision:** Daosheng Li, Gang Hou.

**Validation:** Daosheng Li, Gang Hou.

**Visualization:** Gang Hou.

**Writing – original draft:** Min Lin.

**Writing – review & editing:** Xiaomei Li.
